# *Notes from the Field:* Increase in Pediatric Intracranial Infections During the COVID-19 Pandemic — Eight Pediatric Hospitals, United States, March 2020–March 2022

**DOI:** 10.15585/mmwr.mm7131a4

**Published:** 2022-08-05

**Authors:** Daliya Khuon, Sara Ogrin, Julie Engels, Aileen Aldrich, Rosemary M. Olivero

**Affiliations:** ^1^Pediatric Infectious Diseases, Helen DeVos Children’s Hospital of Spectrum Health, Grand Rapids, Michigan; ^2^Department of Pediatrics and Human Development, College of Human Medicine, Michigan State University, Grand Rapids, Michigan; ^3^Department of Pharmacy, Helen DeVos Children’s Hospital of Spectrum Health, Grand Rapids, Michigan; ^4^Spectrum Health Hospital Group, Grand Rapids, Michigan; ^5^Department of Pediatrics, College of Osteopathic Medicine, Michigan State University, Grand Rapids, Michigan.

During the first 2 years of the U.S. COVID-19 pandemic, pediatric centers anecdotally reported increased rates of intracranial bacterial infections, many of which were diagnosed during or immediately after an infection with SARS-CoV-2, the virus that causes COVID-19 (*1*,*2*). Although intracranial bacterial infections occur as a rare complication of partially treated or untreated bacterial rhinosinusitis in adolescents as well as mastoiditis in children of all ages (*3*), a 236% increase in cases among children was observed at a Michigan children’s hospital (Aldrich A and Ogrin S, Helen DeVos Children’s Hospital of Spectrum Health, unpublished data, 2022). Most of these cases were in infants and children aged <12 years and associated with a diversity of identified pathogens, including a range of *Streptococcus* species with more severe disease requiring extended intensive care unit admission and multiple surgical interventions; many of the cases had recent or concurrent SARS-CoV-2 infection. To ascertain whether a similar trend occurred nationally during the first 2 years of the COVID-19 pandemic, a survey was conducted through the Emergency Infections Network (EIN) of the Infectious Diseases Society of America, a provider-based network of approximately 2,800 infectious diseases specialists primarily based in North America. The initial survey was sent to all EIN participants in February 2022 and queried respondents about whether they had observed an increase in intracranial infections or an increase in invasive *Streptococcus* spp. infections in patients aged ≤18 years during the first 2 years of the COVID-19 pandemic, irrespective of a recent or concurrent COVID-19 infection. The initial survey included 109 respondents, 47 (43%) of whom reported observing an increase in intracranial infections. A follow-up survey was conducted among 64 EIN respondents who expressed interest in further participation, eight of whom were able to query their electronic medical records to determine case rates before the pandemic (January 1, 2018–January 1, 2020) and during the early pandemic (March 1, 2020–March 1, 2022) using *International Classification of Diseases, Tenth Revision* codes (Supplementary Box, https://stacks.cdc.gov/view/cdc/119620). Data were provided by eight institutions, representing all four U.S. Census Bureau regions (Northeast, Midwest, South, and West). Descriptive statistics, including number of diagnoses by infection type, were used (Supplementary Table, https://stacks.cdc.gov/view/cdc/119803). Percent change was calculated for each infection category for each institution, and mean percent change was calculated across institutions. Percent change was used rather than raw numbers because of the wide variability in numbers of cases among institutions. Statistical significance could not be calculated because of the low number of survey respondents. No demographic or COVID-19 vaccination data were collected on individual patients. This activity was reviewed by CDC and was conducted with applicable federal law and CDC policy.*

During the early COVID-19 pandemic, isolated intracranial abscess increased in the participating institutions by a mean of 100.9% (SD = 133%), and sinusitis complicated by intracranial abscess increased by a mean of 76.7% (SD = 97%). Orbital cellulitis, sinusitis, and mastoiditis all decreased on average by 14.5% (SD = 31%), 31.9% (SD = 17%), and 24.7% (SD = 31%), respectively (Figure). Mastoiditis complicated by intracranial abscess decreased by 116.7% (SD = 96%).

**FIGURE:**
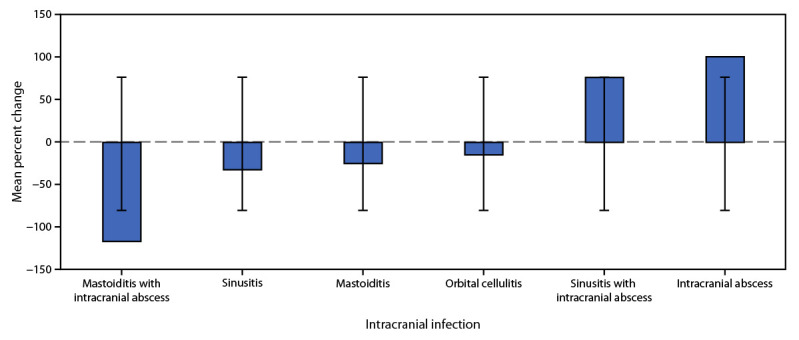
Mean percent change* in number of intracranial bacterial infections^†^ among children and adolescents aged ≤18 years from before the COVID-19 pandemic through the early pandemic — eight U.S. children’s hospitals, January 2018–March 2022 * Mean percent change was calculated across participating institutions for each infection type from the prepandemic (January 1, 2018–January 1, 2020) to the early pandemic (March 1, 2020–March 1, 2022) periods. SD shown by error bars. ^†^ Intracranial abscess count does not include counts from sinusitis with intracranial abscess or mastoiditis with intracranial abscess.

The findings in this report are subject to at least three limitations. Because participation in the EIN surveys was voluntary, the results are not representative of intracranial infections throughout the United States. Second, the response rate was low, and response bias possibly affected the findings. Finally, only limited data were collected, and the cross-sectional nature of this analysis precludes inference of causation.

On June 8, 2022, CDC asked health care providers and health departments to report the occurrence of brain abscess, epidural empyema, or subdural empyema in persons aged ≤18 years without a previous history of neurosurgical procedures or head trauma, hospitalized on or after June 1, 2021, and to retain associated clinical specimens and isolates. To report possible cases,^†^ health care providers should contact their health department and email CDC (CDCStrepInquiry@cdc.gov).

This initial investigation suggests a possible increase in some forms of intracranial infections in persons aged ≤18 years living in the United States during March 2020–March 2022, coinciding with the first 2 years of the COVID-19 pandemic. Further characterization of affected patients, disease course, temporal association with COVID-19 infection (as well as predominant variants circulating at the time of diagnosis), microbiology of cases, and morbidity and mortality associated with this observation are needed, because the factors causing this possible increase in intracranial infections are not fully understood at this time. Intracranial infections require prompt and intensive medical management; therefore, defining the pathogenesis, relation to SARS-CoV-2 infection, and other risk factors, can both raise public awareness and help medical providers diagnose and appropriately manage affected patients. 

## Footnotes

† CDC is asking health care providers and health departments to report the occurrence of brain abscess, epidural empyema, or subdural empyema in persons aged ≤18 years without a previous history of neurosurgical procedures or head trauma, hospitalized on or after June 1, 2021. Health care providers and health departments are encouraged to report cases that are 1) associated with streptococcal infection, 2) because of other bacterial pathogens, 3) polymicrobial, or 4) have no pathogen identified.
